# Highly efficient photon detection systems for noble liquid detectors based on perovskite quantum dots

**DOI:** 10.1038/s41598-020-73437-x

**Published:** 2020-10-09

**Authors:** Amlan Datta, Biplob Barman, Stephen Magill, Shariar Motakef

**Affiliations:** 1 CapeSym, Inc., 6 Huron Drive, Natick, MA 01760 USA; 2 University of Michigan-Flint, 303 E Kearsley St, Flint, MI 48502 USA; 3 Argonne National Laboratory, 9700 S Cass Ave, Lemont, IL 60439 USA

**Keywords:** Materials science, Nanoscience and technology, Optics and photonics

## Abstract

Wavelength shifting photon detection systems (PDS) are the critical functioning components in noble liquid detectors used for high energy physics (HEP) experiments and dark matter search. The vacuum ultraviolet (VUV) scintillation light emitted by these Liquid argon (LAr) and liquid Xenon (LXe) detectors are shifted to higher wavelengths resulting in its efficient detection using the state-of-the-art photodetectors such as silicon photomultipliers (SiPM). The currently used organic wavelength shifting materials [such as 1,1,4,4 Tetraphenyl Butadiene (TPB)] have several disadvantages and are unreliable for longterm use. In this study, we demonstrate the application of the inorganic perovskite cesium lead bromide (CsPbBr_3_) quantum dots (QDs) as highly efficient wavelength shifters. The absolute photoluminescence quantum yield of the PDS fabricated using these QDs exceeds 70%. CsPbBr_3_-based PDS demonstrated an enhancement in the SiPM signal enhancement by up to 3 times when compared to a 3 µm-thick TPB-based PDS. The emission spectrum from the QDs was optimized to match the highest quantum efficiency region of the SiPMs. In addition, we have demonstrated the deposition of the QD-based wavelength shifting material on a large area PDS substrate using low capital cost and widely scalable solution-based techniques providing a pathway appropriate for meter-scale PDS fabrication and widespread use for other wavelength shifting applications.

## Introduction

Noble liquid detectors are being used and considered for a variety of applications involving precise particle tracking, calorimetry and spectroscopy in high energy physics, γ-ray astronomy, neutrinoless ββ-decay, and medical imaging. These include detectors that are currently under development for dark matter search and neutrino experiments. A major requirement for these experiments is the detection of the vacuum ultraviolet (VUV) scintillation light with high efficiency to enable three-dimensional spatial localization of the events. The incoming particles in these experiments deposit their energy through three primary mechanisms: (a) scintillation from de-excitation of dimers in the noble liquid, (b) creation of free electrons through ionization, and (c) thermal losses, with the collection of scintillation photons as the primary time detection method. Scintillation from the excited dimers is produced by two processes: (i) recombination of recoiling electrons with noble element ions forming excited dimers in the noble liquid, and (ii) formation of excited dimers at gas/liquid interface due to collision with high energy electrons in the gas. The former is the only process in single phase (SP) detectors, and both occur in dual phase (DP) detectors. For both DP and SP detectors, pulse shape discrimination of the scintillation signal from the first process provides information on the electron and nuclear recoil ratios. For DP detectors, the ratio of scintillation responses from the two processes provides additional information to discriminate between the electron and nuclear recoils.

**Figure 1:**
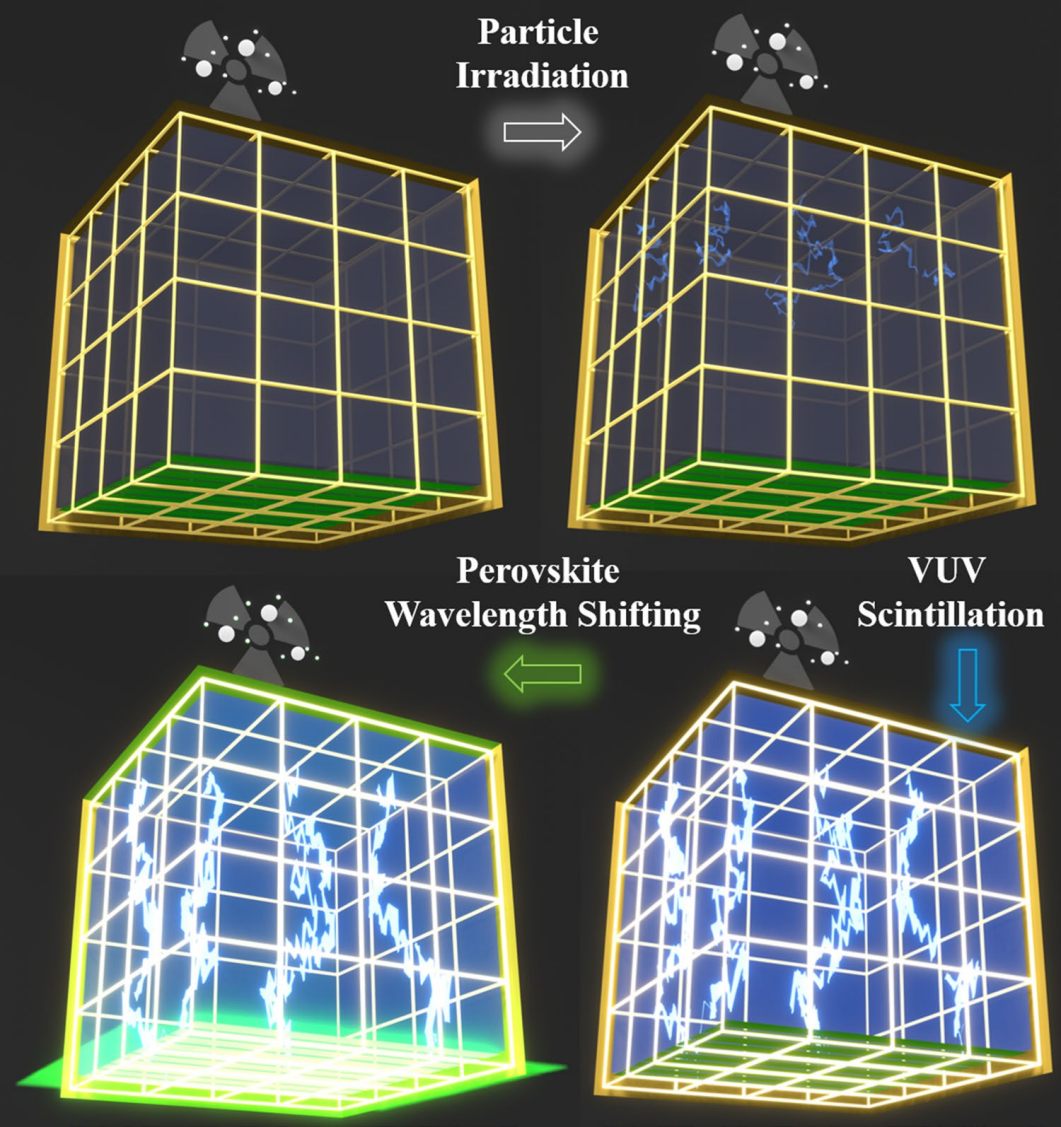
These figures illustrate how rare particle events are identified by noble liquid detectors. The incoming particle creates VUV scintillation (represented by blue light) inside the noble liquids such as LAr or LXe. These VUV photons are shifted to higher wavelengths, and are detected by present day photodetectors (such as SiPMs). The overall efficiency of the detection mechanism depends primarily on the wavelength shifting efficiency.

The relatively high scintillation light output with exceptional optical transmission over long distances in noble liquid detectors make the scintillation process an excellent method for event-triggering and determining the t_0_ for non-beam related events (such as muon tracking and tagging), and for performing background rejection. For example, the light output from LAr detectors is about 20 k photons/MeV under bias, and the emitted 128 nm VUV photons have a Rayleigh scattering length of 66 ± 3 cm and absorption length of > 200 cm in LAr^1^. However, the short wavelength of the scintillation photons (128 nm for LAr and 178 nm for LXe) does not match the spectral absorption band of current photosensors and must be converted into longer wavelengths for efficient detection. This is achieved by using wavelength shifters (WLS) between the outer boundary of the noble liquid detector and the photosensors. Figure 1 summarizes the scintillation light generation and collection process dynamics in these detectors. Supplementary Video 1 shows the animation of the light generation and wavelength shifting process. The accuracy and fidelity of the signal at the photosensor are uniquely determined by the quality and characteristics of the WLS material and design.

Currently, the following configurations are used for collection and wavelength shifting of the LAr scintillation photons to longer wavelengths: SiPMs with WLS and light guides and PMTs with WLS coatings. Two arrangements of the photon detection systems (PDS) used in the ProtoDUNE (DUNE stands for Deep Underground Neutrino Experiment ) single-phase time projection chamber are shown in References^2,3^. In these PDS, the incident 128 nm light is absorbed by a WLS layer, is wavelength shifted to 430 nm, and is waveguided to the SiPM at 430 nm^2^ or in the same arrangement, the waveguide is also made of WLS material and the light is further wavelength shifted to 490 nm as it travels to the SiPM^3^. The third type of detector under development (ARAPUCA) uses WLS plates but employs a different mechanism for transmitting light to the SiPMs^4^. In all of these systems, 1,1,4,4 Tetraphenyl Butadiene (TPB) is used as the wavelength shifting chemical, either applied as a coating or diffused in a plastic host. The PDS in the ProtoDUNE dual-phase detector consists of 36 8-inch cryogenic PMTs. TPB is either directly coated onto the PMTs or is deposited on a plate that is placed on top of the PMT.

TPB is the most commonly used WLS material^5–11^ for the VUV scintillation light. Its emission wavelength (around 430) nm matches the high quantum efficiency wavelength range of many standard PMTs and SiPMs^12^. However, it has several major shortcomings that include deterioration of TPB-based WLS coatings over time, delayed light emission, and low efficiency. The deterioration phenomena mainly involve photodegradation and emanation of TPB layers when submerged in LXe and LAr TPCs. When TPB is exposed to UV light, benzophenone is released. This is a well-known UV blocking photo-initiator compound that is hypothesized to be the reason behind the performance degradation observed in TPB coatings^13,14^. The emanation of the TPB thin film layers in the cryogenic noble liquids is also a significant problem for long-term applications. It has been shown that the TPB WLS film thickness decreases by 24% after immersion in LXe within only 20 h^15^. A recent study has shown a detectable concentration of TPB in LAr over a 24-h period, corresponding to tens of parts per billion in argon by mass, which in turn produces a wavelength shifting effect in the LAr scintillation^16^. Although the severity of these effects depends on several factors, including liquid flow rate, coating preparation techniques, fill procedure, filtration method, etc., the aberrations introduced by TPB WLS films in the TPC-based experiments are well documented. Reference^17^ is the only study we have found refuting the importance of performance degradation of TPB-based WLS coatings. In the LAr detector, roughly 1/4th of the photons emitted have a lifetime of 6 ns and the rest 1100–1600 ns^1^. The relative abundance of the fast and slow decay components of the LAr scintillation is often used to identify the particle type. The use of TPB degrades this feature of LAr since part of the prompt light is delayed, distorting the distinction between the two populations. When TPB is excited by 127 nm VUV photons from LAr, it reemits photons not only with a very short decay time (~ 1nsec) but also with slower ones due to triplet states de-excitations. This feature of TPB is responsible for the anomalies in LAr scintillation light reported since the seventies^18^. The forward conversion efficiency, i.e., the ratio of photons incident on SiPM to the low wavelength photons coming from the noble liquid source, of the WLS layer is estimated to be only 10%^19^.

In this study, the performance of the WLS-based PDS was significantly improved by using fully inorganic CsPbBr_3_ perovskite Quantum Dots (QD) as the WLS material. The inorganic perovskite QDs exhibit high chemical stability, single component ultra-fast decay time, and absolute quantum efficiencies close to 90%. Colloidal semiconductor nanocrystals (typically 2–20 nm in dimensions), widely referred to as quantum dots (QDs), are being intensively studied as the next generation of optoelectronic materials^20–23^. The semiconductor QDs combine the enhancement of the optical properties due to the quantum-scale effects with the solution processability for ease of incorporation and dispersal into a variety of substrates. To date, the best developed optoelectronic QDs in terms of size, shape, and composition are binary and multinary (ternary, quaternary) metal chalcogenide QDs^20,24–26^. Applications of these QDs include bioimaging, lighting, displays, solar cells, photodetectors, and lasers^27,28^. They are present in commercial lighting and display products. The most frequently investigated system is cadmium-based chalcogenides, which dominated the QD space for decades, while some issues, such as high reaction temperature and surface passivation (shelling), hindered their scaled production for practical applications. Application of these chalcogenide QDs are new in the field of WLS in noble liquid detectors, but the initial results have proven the feasibility of using QDs as WLS materials viable^29,30^. CsPbBr_3_ perovskite QDs exhibit significantly better performance than these traditional QDs.

The as-synthesized colloidal CsPbBr_3_ QDs are cubic in shape with cubic perovskite crystal structure^31^. The reduced nanometer-size, required for stabilizing the cubic phase exhibits quantum-size effects i.e., higher bandgap energies due to quantum confinement with energy ΔE = ℏ^2^π^2^/2 m*a^2^ where a and m* are the particle radius and the reduced mass of the exciton. The calculated effective Bohr diameters of Wannier-Mott excitons and the binding energies for CsPbBr_3_ are 7 nm and 40 meV, respectively^31^. CsPbBr_3_ QDs are highly ionic and thus follow an ordered crystallinity and stability. This is different from other multinary chalcogenides that exhibit significant disorder and inhomogeneity in the distribution of cations and anions owing to the small difference between the cationic and anionic site sizes. Even without surface shelling, photoluminescence QYs (PLQYs) as high as 90% have been achieved with perovskite QDs^31–34^. As we will show in this study, at cryogenic temperatures, the QY increases several folds. At room temperature, the QDs have radiative lifetimes of 1–29 ns, which decreases to below a nanosecond at cryogenic temperatures^31,35^. Unlike other QD systems, the electronic properties of CsPbBr_3_ QDs are highly tolerant to the material’s defects and surface states^36^. In conventional semiconductors, the bandgap is formed between bonding (σ) and antibonding (σ*) orbitals. Point defects or dangling bonds emerge as weak or non-bonding states within the bandgap. In CsPbBr_3_ QDs, the shallow character of the vacancy-related states is due to the antibonding nature of the valence band maxima and spin–orbit effects in the conduction band^37^. Thus, point defects and dangling bonds do not strongly influence the radiative recombination and other optical properties of the QDs, resulting in high PLQYs. CsPbBr_3_ QDs not only allow compositional bandgap engineering using halide anion doping but owing to the exciton Bohr diameter of up to 12 nm, they also exhibit size-tunability of their bandgap energies corresponding to the entire visible spectral range of 410 − 700 nm^31^. In this study, we have obtained high PLQY from the PDS by optimizing the emission wavelengths of the CsPbBr_3_ QDs.

## Results and discussion

**Figure 2:**
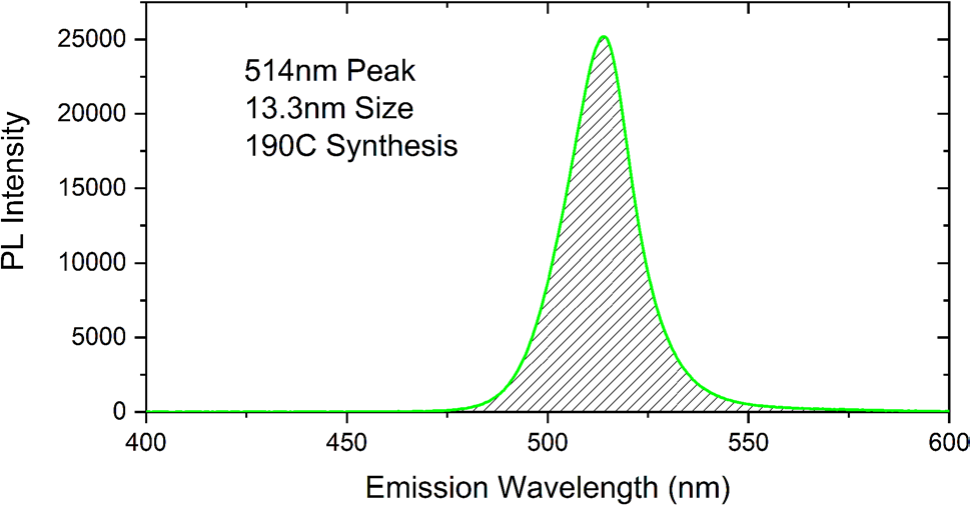
PL Emission spectrum of CsPbBr_3_ QDs with 405 nm excitation and an output power of 0.7 mW.

The characterization studies reported in this section include both the as-synthesized CsPbBr_3_ QDs and the PDS fabricated on different substrates. The main focus of the results are on the performance of the PDS fabricated using the QDs. Figure 2 shows the typical room-temperature PL emission spectrum of the CsPbBr_3_ QDs excited with 405 nm laser. The PLQY of the CsPbBr_3_ QDs fabricated using different techniques were estimated using an integrated sphere. Measurements of the integrating spheres are validated with two standard samples, quinine sulfate, and rhodamine, which are accepted PLQY reference materials. For all the PLQY measurements of the QDs, the concentration was chosen to be ~ 10 mg/ml. For TPB PLQY measurements, concentrations of 10 mg/ml and 50 mg/ml was used in toluene. The PLQY values of the QDs and TPB are listed in Table 1.

**Table 1:** PLQY, emission wavelength, and chromaticity of CsPbBr_3_ QDs with a concentration of 10 mg/ml fabricated using different techniques and TPB in different concentrations.

Chemical composition	Synthesis procedure, temperature	Typical PLQY (%)	Emission wavelength (nm)	Chromaticity (CIE1976)
CsPbBr_3_ QD	OAm synthesis, 155 ºC	70.42	490	(0.0299, 0.5113)
	OAm synthesis, 180 ºC	62.98	511	(0.0185, 0.5628)
	OAm synthesis, 190 ºC	62.36	518	(0.0300, 0.5696)
	DSA synthesis, 120 ºC	39.51	517	(0.0345, 0.5807)
	DSA synthesis, 120 ºC, Excess Pb	28.33	514	(0.0334, 0.5838)
TPB	Toluene Solution, 10 mg/ml	3.89	432	(0.1796, 0.1370)
	Toluene Solution, 50 mg/ml	4.62	432	(0.1773, 0.1417)

**Figure 3:**
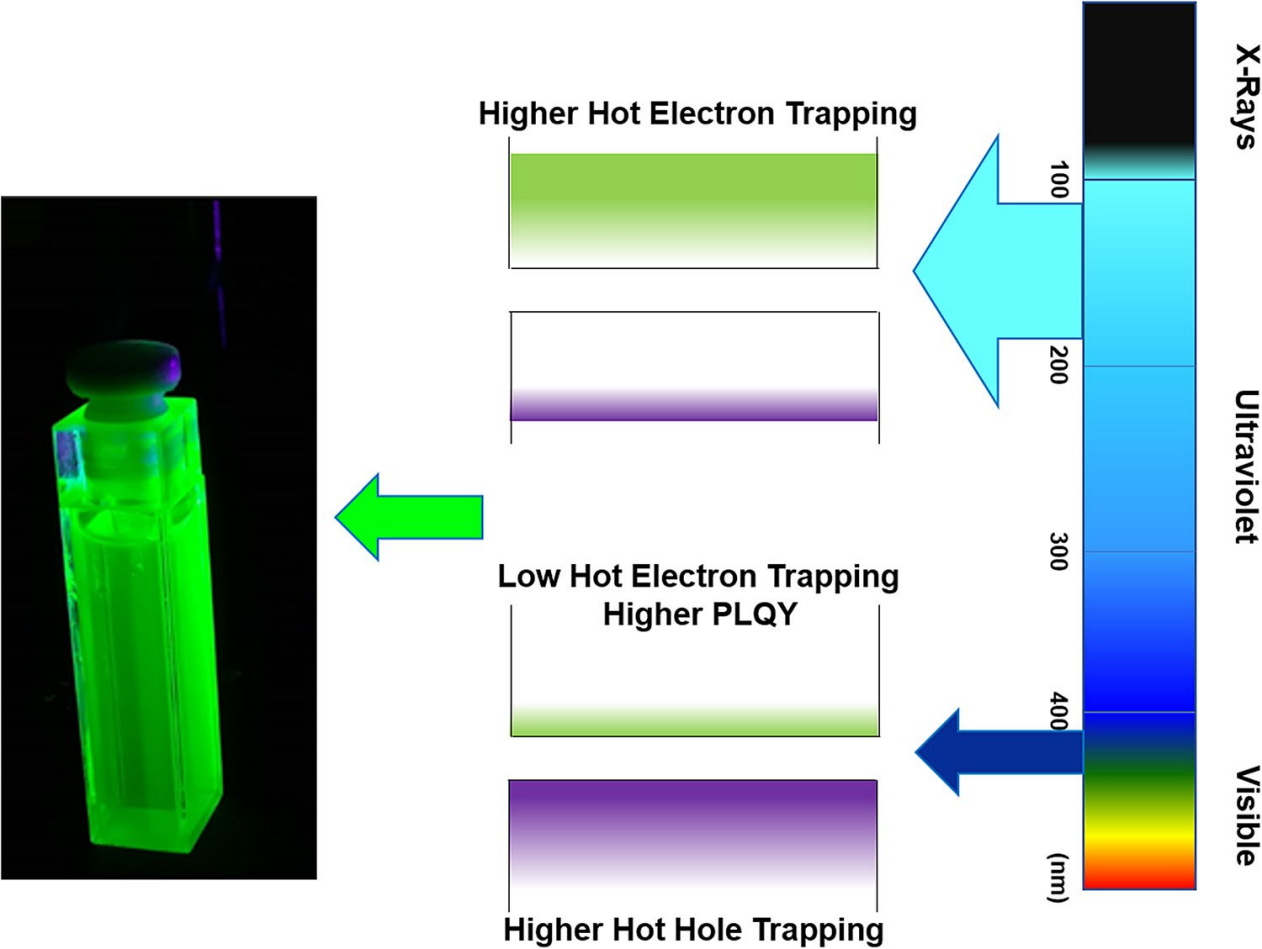
Schematic diagram for excitation wavelength dependent electron and hole trapping mechanism in CsPbBr_3_ QD system.

The lower PLQY values of the DSA synthesis are due to unoptimized surface ligand properties, which result in trapping channels for hot carriers resulting in photoluminescence quenching. Due to the lower excitation energy, the probability of non-radiative energy transfer between the hot exciton and vibrational modes of the ligands is higher. Also, the ratio of initial metal precursors have a significant effect on the QD photoluminescence properties and is less forgiving than the amine ligand-based (such as OAm) processes. The PLQY of QDs is significantly dependent on the excitation wavelength. This dependence has been discussed for CdSe based QDs^38,39^. With decreasing excitation wavelength, the PLQY generally decreases. The main reason for this is the excitation energy-dependent changes in the hot carrier trapping, if the excitation is far from the band edges, the hot hole trapping is lower, but the hot electron trapping increases from the shallow traps created by QD surface defects or ligand-related defects. This suggests that for a low CsPbBr_3_ bandgap (~ 539 nm) system, lower the excitation wavelength higher is the probability for non-radiative recombination of the majority hot-electron carriers. So, for 220 nm excitation, the probability of hot electron trapping increases significantly, resulting in a lower PLQY of 70.42%. On the contrary, when the excitation is closer to the band-edge, i.e., the excitation wavelength is higher, the hot electron trapping probability decreases. However, electron traps innate to the QD system still exists. These trapped electrons, along with the higher number of trapped holes due to the band edge excitation, creates excitons that increases the PLQY of such a system (Fig. 3). For the CsPbBr_3_ QDs in this study, with 410 nm excitation, PLQY of over 86% was obtained. So, for a 46% decrease in the excitation wavelength, the PLQY value decreased by 18.1%. With a further decrease in the excitation wavelength for LAr (− 43%), we expect the PLQY to decrease to somewhere close to 58%. However, it must be noted that lowering of temperature significantly passivates these hot carriers traps, resulting in significantly higher PLQY. As we will see from the low-temperature PL results, shown later in this section, PLQY can increase by 200% at LAr temperatures.

We have also tested QDs with different stoichiometries, such as CsPbBr_1.5_Cl_1.5_ and CsPbCl_3_. The PLQY and emission wavelengths for these QDs are listed in table S1. The emission spectra of these QDs are shown in Figures S1 and S2. The CsPbBr_3_ QDs with the highest PLQY were deposited on various substrates for the fabrication of the PDS. The color gamut for the OAm and DSA synthesized CsPbBr_3_ QDs are shown in Figures S3 and S4. The SiPM output results for each type of substrates are summarized in the sections below.

### UV quartz

**Figure 4:**
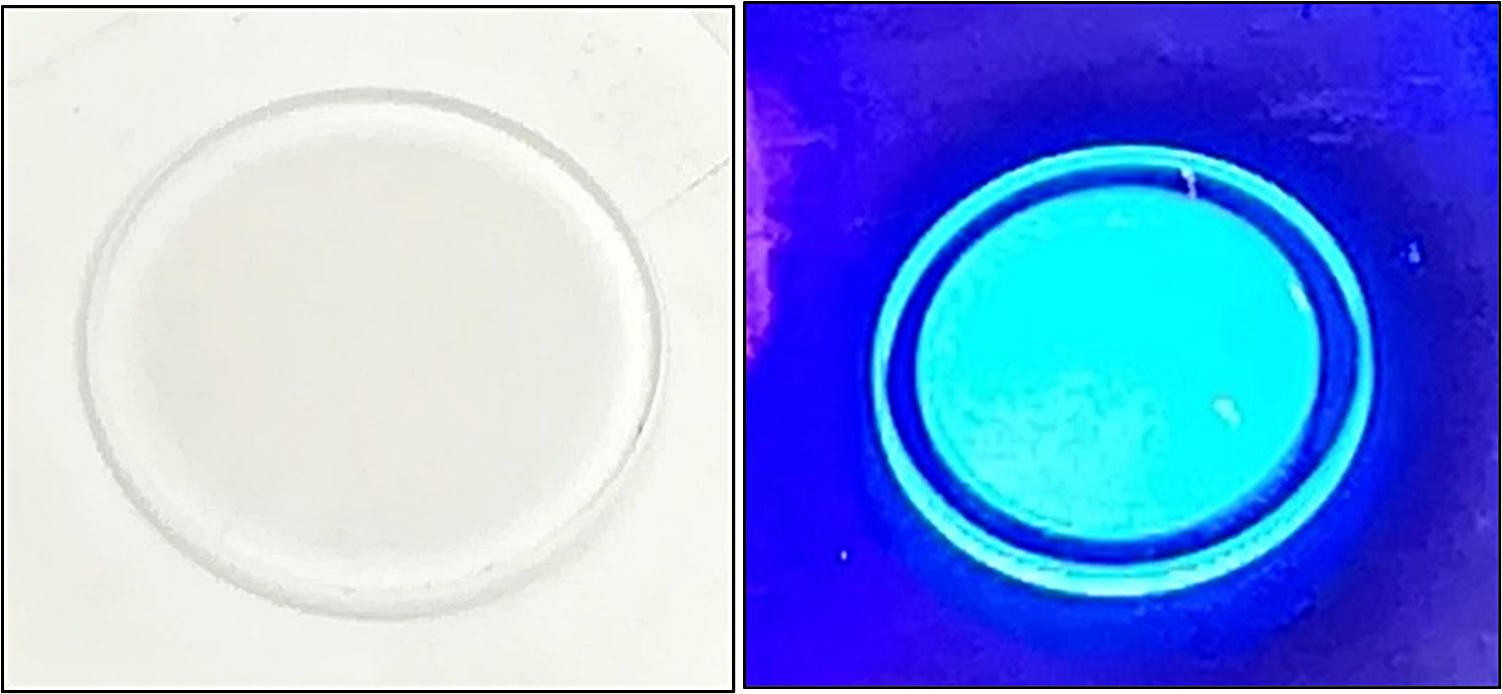
(Left) 3 µm-thick TPB deposited on the silica substrate, (right) Blue emission from TPB under UV irradiation.

We used 12.5 mm diameter UV-grade fused silica substrates from Esco optics as substrates for the WLS PDS. CsPbBr_3_ QDs were deposited on these substrates using spin coating. The coated substrate was dried under vacuum for 15 min and in a box furnace at 50 °C for another 15 min. Once dry, the discs were attached to the SiPM using an optical gel suitable for transmission of wavelengths above 300 nm. Figure S5 shows an uncoated silica disc for the control experiment and the CsPbBr_3_-based PDS attached to the SiPM. Along with the control samples with uncoated silica discs on the SiPM (Figure S5), we fabricated TPB-based reference PDSs. TPB was deposited using a thermal evaporation technique inside the glovebox. Figure S6 shows the TPB source material used for the thermal evaporation. Figure 4 (left) shows the TPB coated silica PDS under reflected light, and under UV irradiation, Fig. 4 (right). Two thicknesses of 3 µm and 30 µm of TPB were deposited with the former in the range of film thickness used in noble liquid detectors such as in MiniCLEAN, NEXT, and DEAP-3600 experiments^40,41^, and the latter to demonstrate the loss of transmission at high thicknesses of TPB.

In order to get a quantitative estimation about the WLS sensitivity of the PDS, the SiPM voltage output needs to be weighed using various factors:

1. Change in excitation intensity with the wavelength,
2. Transmission properties of the PDS substrate, and
3. Transmission properties of the excitation fiber.

**Figure 5:**
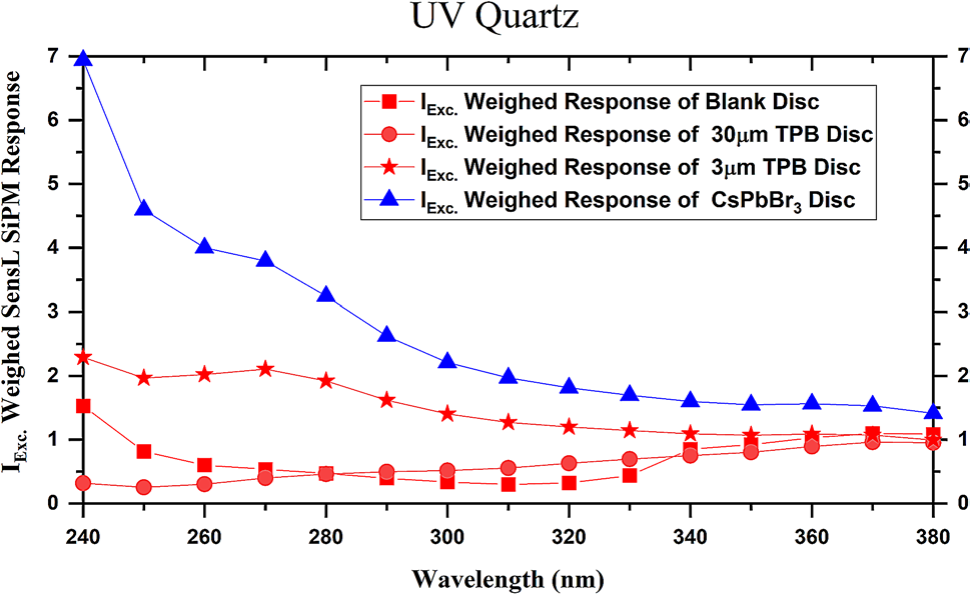
Plots for weighed normalized WLS response of various PDS versus excitation wavelength. The 3 µm-thick TPB is similar to detectors currently used in liquid noble detectors.

The second and third factors are negligible for most of the substrates and wavelength range we are interested in. However, for the Xenon source, excitation intensity at the lower wavelengths (below 350 nm) decreases significantly. The normalized values of these weighed responses are shown in Fig. 5. In these weighed responses, it is clear that at 240 nm excitation, there is a signal enhancement of about 4.6 times. Also, with respect to 30 µm TPB the CsPbBr_3_ QD-coated PDS shows a 17-times improvement. The effect of the WLS function is clear in these plots. It is clear that effectiveness of the WLS functionality increases in the following order: Blank < PDS ~ 30 µm-thick TPB < 3 µm-thick TPB < CsPbBr_3_ QD.

### Acrylic

**Figure 6:**
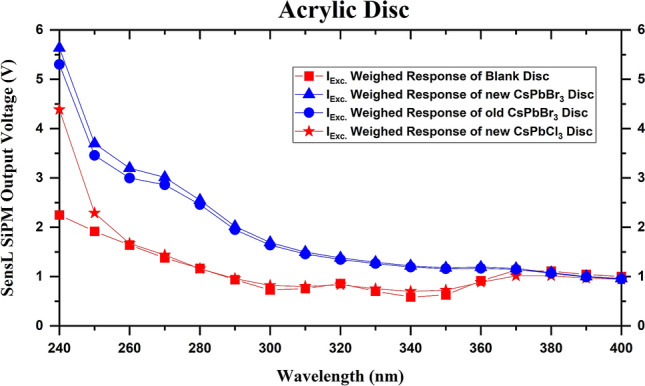
Plots for weighed normalized WLS response of various acrylic-based PDS versus excitation wavelength.

PDS based on 25.4 mm diameter acrylic discs were fabricated using drop-casting. The CsPbBr_3_ QD colloidal solution in toluene was used as the precursor solution. The drying process involved 2 h of vacuum treatment and 1 h of baking. Figure S7 shows the uncoated and PDS discs under ambient and UV light. The SiPM output and the weighed responses of a blank acrylic disc, PDS with new and “old” CsPbBr_3_ QD-based, a CsPbCl_3_ QD-based PDS are shown in Fig. 6. The “old” CsPbBr_3_ QD refers to CsPbBr_3_ QDs stored inside the glovebox for 1 month. These QDs were used to see the short-term effect of storage in controlled environments (such as inside a noble liquid detector). Due to the low PLQY of the CsPbCl_3_ PDS, even with an emission wavelength that matched the highest PDE of the SiPM, its response was weak and will not be a good WLS material for the wavelengths tested here. However, it shows an increase in the response at lower wavelengths and may continue to improve for wavelengths below 240 nm. We have also tested several post-synthetic treatments to enhance the PLQY of the CsPbCl_3_ QDs using YCl_3_ and CdCl_2_ passivation^42–44^ but did not see any appreciable repeatable enhancement (Table S1). Reliable PLQY enhancement of the blue QDs needs further research and was out of the scope of our work. Thus, our study was focused on the PDS based on CsPbBr_3_ QDs, which showed excellent acrylic WLS properties, as evident from Fig. 6.

### UV-blocking plastic sheets

**Figure 7:**
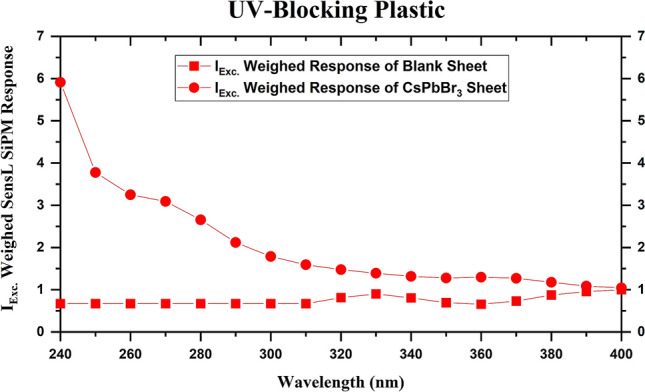
Plots for weighed normalized WLS response of various PDS versus excitation wavelength. The clear evidence of the wavelength shifting effect can be seen here.

UV-blocking plastic sheets block all the wavelengths below 300 nm. As a result of which any response from the SiPM with excitation wavelengths below 300 nm will only be from the CsPbBr_3_ QD WLS. Figure 7 shows the weighed SiPM response of the CsPbBr_3_ QD coated and uncoated UV-blocking sheet PDS. Here, it is clear that the wavelength shifting effect results in a signal gain of more than ten times.

### PMMA light guide

**Figure 8:**
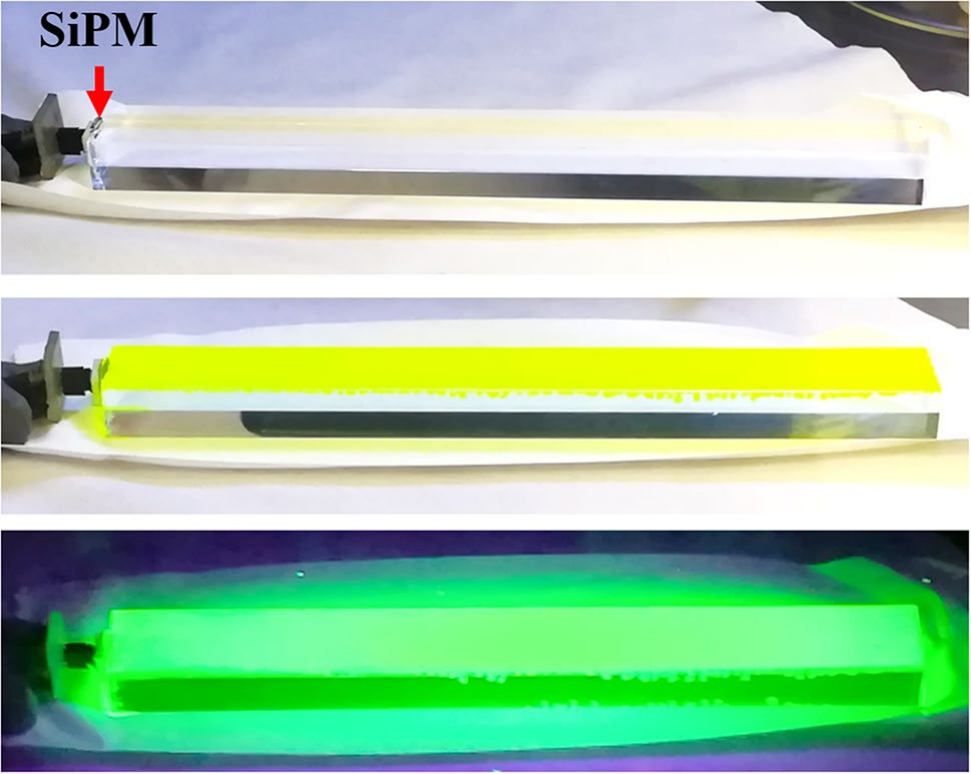
(Top to bottom) 15 cm long PMMA bar polished along all sides, CsPbBr_3_ QD coated PMMA bar under ambient light and UV light.

**Figure 9:**
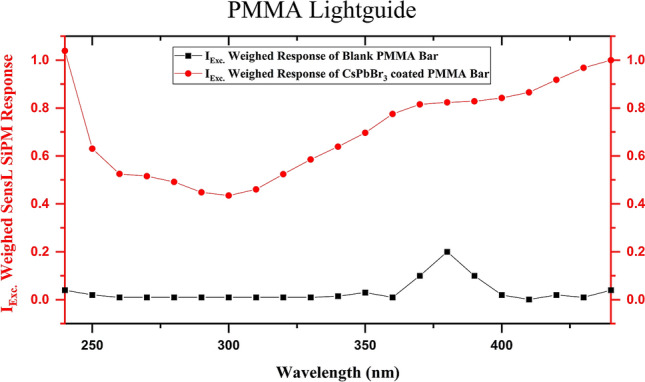
Weighed response of irradiated PMMA bar with and without CsPbBr_3_ QD coating.

As a final step for showing the feasibility of the proposed design, we fabricated a PDS precisely 1/13th of the length of the PDS currently being used for ProtoDUNE-SP experiments (Fig. 8). The 15 cm long PMMA light guide was polished on all sides to a mirror finish. The SiPM is attached to the far end of the light guide. The PDS was fabricated from PMMA due to its excellent light transmission properties at 514 nm, as listed in Table 2. In addition to PMMA, we also tested light guides fabricated using polystyrene (PS). But as can be expected from the extinction coefficient (κ) values and the calculated absorption coefficient (α) parameters from the formula $$\alpha = {\raise0.7ex\hbox{${4\pi \kappa }$} \!\mathord{\left/ {\vphantom {{4\pi \kappa } \lambda }}\right.\kern-\nulldelimiterspace} \!\lower0.7ex\hbox{$\lambda $}}.$$, we were not able to get a high signal from the SiPM for PS based PDS. CsPbBr_3_ QDs were coated onto it using a spray coating technique. Other prototypes were also fabricated using dip coating, but the best properties were obtained from the spray coating technique. However, it must be noted that in the liquid noble gas detectors, the dip-coated bars will have a better performance due to the omnidirectional VUV excitation. The PDS’s performance with a SIPM was excellent using 220 nm excitation and showed that the wavelength shifting process produces significant improvement in the SiPM signal. Figure 9 compares the weighed SiPM response of this large PDS with and without the QD WLS layer. The effect of total internal reflection inside the light guide is also evident. Here, the 200 µm diameter excitation optical fiber was placed at the farthest end of the light guide to test the limiting case scenario. Using a larger diameter optical fiber and moving this excitation source closer to the SiPM yields a significantly higher SiPM response. This concludes the feasibility of the proposed approach for the application of WLS.

**Table 2:** Optical constants of PMMA and PS as light guides at the QD emission wavelength of 514 nm.

Light guide material	Refractive index	Extinction coefficient	Absorption coefficient	Chromatic dispersion	Group index
PMMA	1.489	2.164E−7	0.053 cm^−1^	− 0.07 µm^−1^	1.525
PS	1.598	5.960E−7	0.146 cm^−1^	− 0.14 µm^−1^	1.671

**Figure 10:**
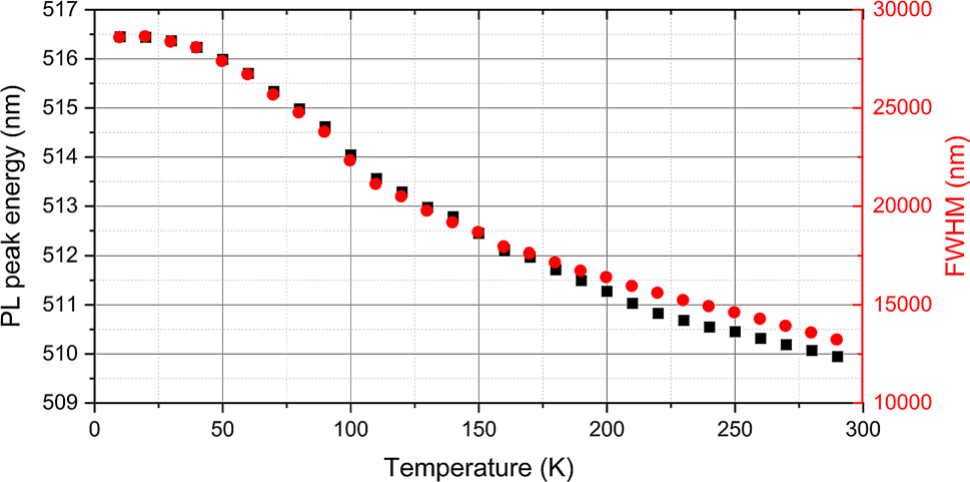
Shifts in the PL emission spectra peaks and FWHM with temperature.

**Figure 11:**
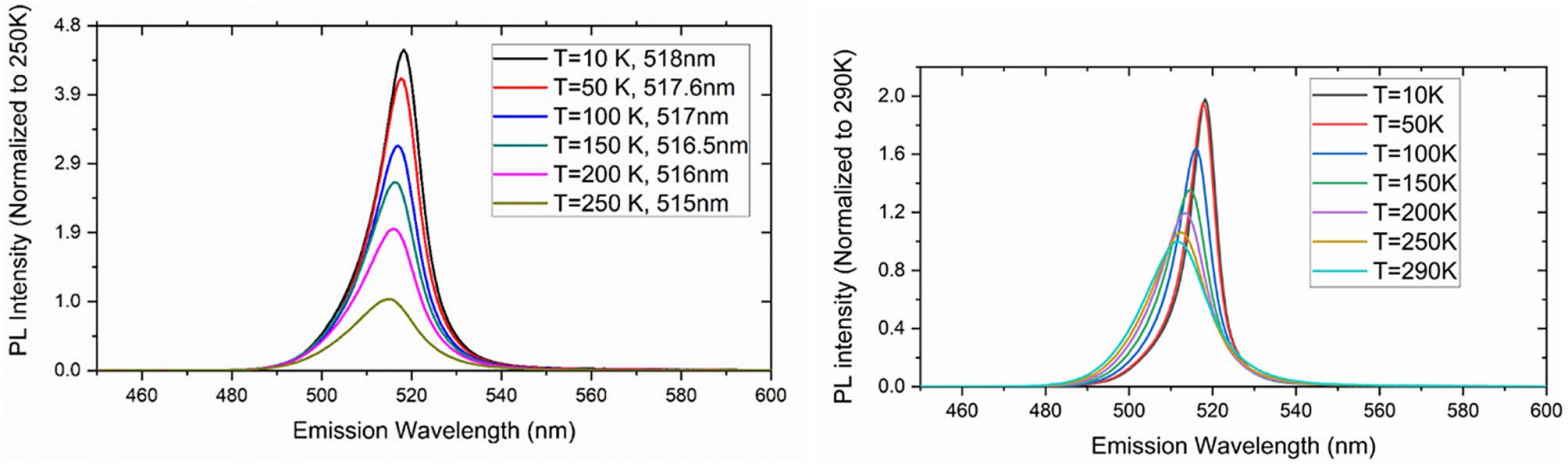
Changes in the PL emission spectra with temperature for OAm (left) and DSA (right) synthesized CsPbBr_3_.

In addition to the above-mentioned room-temperature experiments, we tested the low-temperature performance of the CsPbBr_3_ QDs to determine its performance in liquid noble gas detectors. Figure 10 shows the change in the peak positions and the FWHM of the emission for temperatures between 10 and 290 K. Figure 11 shows the PL spectra of the OAm and DSA synthesized CsPbBr_3_ QDs at various temperatures normalized to 250 K and 290 K. It is clear that with decreasing temperature, the relative intensity of the emission, and hence the PLQY of the QDs increases by more than 4.5 times. This demonstrates the feasibility of using these QDs at lower temperatures, such as 87 K for liquid argon and 165 K for liquid Xenon.

## Conclusions

In this study, we have demonstrated the feasibility of CsPbX_3_ QDs as an excellent WLS with high photoluminescence quantum yields (PLQYs), using excitation as low as 220 nm. These results show significantly enhanced signals from SiPM-based photon detection systems (PDS) compared to TPB. Several QD fabrication parameters and techniques were investigated to obtain the optimal QD based WLS. The chemical composition of the QDs was varied to obtain the highest PLQY and PDS response. We concluded that CsPbBr_3_ QDs synthesized at 155ºC using Oleylamine (OAm) hot injection synthesis technique gives the best PLQY and hence, best PDS performance. PLQYs as high as 70% were obtained using CsPbBr_3_ QDs in a colloidal solution as well as in PDS. The performance of the QDs was stable for more than a month inside an argon glove box. A CsPbBr_3_-based PDS demonstrated SiPM signal enhancement of up to 3 times when compared to a 3 µm-thick TPB-based PDS—3 µm TPB film thickness is comparable to the thickness of detectors used in MiniCLEAN, NEXT, and DEAP-3600 experiments. We also showed that at temperatures as low as 10 K, the PLQY of CsPbBr_3_ QDs increased by a factor of 2 to 5, giving a positive outlook for application at noble liquid temperatures. A large PDS with dimensions in the order of 1/13^th^ of the PDS used in ProtoDUNE-SP experiment was fabricated based on SiPMs, and its functionality as a WLS was demonstrated using 220 nm excitation.

## Experimental section

### OAm synthesis of CsPbBr_3_ QDs

OAm synthesis of CsPbBr_3_ QDs was performed by injecting cesium oleate solution to the lead bromide precursor solution. Cs_2_CO_3_ (Sigma Aldrich 99.9%) was loaded into a 50 ml three neck flask along with octadecene (ODE) (Sigma-Aldrich, 90%) and oleic acid (OA) (Sigma Aldrich, 90%), in an argon glovebox. Two of the necks were closed using rubber septa and the third was attached to a quartz O-ring joint through a valve. After mixing the precursor chemicals, the valve was closed, and the flask was taken out of the glovebox. It was then attached to a Schlenk line capable of pulling vacuum and filling with UHP N_2_. A heater with a magnetic stirrer was placed below the flask. The temperature of the solution was monitored using a Teflon coated K-type thermocouple. It was inserted through the septa of the flask’s left neck. The solution was dried for 1 h at 120 ºC. Then, the flask was subjected to alternating vacuum and N_2_ purge cycles. The Cs-oleate solution was heated to 150 ºC until all Cs_2_CO_3_ reacted with OA. Since Cs-oleate precipitates out of ODE at room-temperature, it has to be preheated to 150 ºC before injection. ODE and PbBr2 (Alfa Aesar, 98%) were loaded into another 50 ml 3-neck flask along with Oleylamine (OLA) (Sigma Aldrich, 90%) and OA in an argon glovebox atmosphere. After complete solubilization of the PbBr2 salt, the temperature was raised to 155–190 ºC (for tuning the QD size) and Cs-oleate solution (0.15 M in ODE, prepared as described above) was quickly injected and, 5 s later, the reaction mixture was cooled by the ice-water bath. CsPbCl_3_ and CsPbBr_1.5_Cl_1.5_ QDs were also fabricated using the same procedure. For CsPbCl3, trioctylphosphine (TOP, Sigma Aldrich, 97%) was added to the precursor solution to solubilize PbCl2. For the fabrication of CsPbBr_1.5_Cl_1.5_, equal quantities of bromide and chloride lead salt precursors were added in the flask.

### DSA synthesis of CsPbBr_3_ QDs

This synthesis technique is also based on the hot injection technique. Cs_2_CO_3_, lead acetate (Pb(OAc)_2_), ODE (10 ml), and DBSA (Sigma Aldrich, 95%) were loaded into a 50 ml three-neck flask and the mixture was dried at 120 ºC using the procedure demonstrated above for one hour. A transparent solution indicates complete reaction and formation of cesium and lead sulfonates. Pre-dissolved toluene solution of the halide precursor such as tetraoctylammonium bromide (TOAB) (Sigma Aldrich, 98%) for CsPbBr_3_ was swiftly injected into the above precursor solution. After 5 s, the reaction mixture was cooled down using a water bath.

### Isolation and purification of CsPbX_3_ QDs

The cold crude solution containing the QDs was poured into 15 ml centrifuge tubes. These solutions were weight balanced and centrifuged at 8000 rpm for 5–10 min, depending on the total volume. After centrifugation, the supernatant was discarded, and the particles were dispersed in hexane or toluene, forming colloidal solutions of the QDs (Figure S10). In some cases, the QDs were washed using ethyl acetate re-precipitation and centrifugation steps. However, the QDs were more stable when the washing step was not performed. The concentration of the QDs was kept at 10 mg/ml.

### PLQY measurement

The wavelength shifting properties of the QDs were analyzed using a Horiba spectrofluorometer. The absolute quantum yields of the QDs and the PDS were calculated using an integrated sphere. For PLQY measurements, the incident excitation beam was tuned to 220 nm with a cutoff filter that blocks all the wavelengths above this.

### Photoluminescence spectroscopy

The sample was placed in a variable temperature closed-cycle optical cryostat with a temperature range 10–350 K. Photoluminescence was excited using a continuous wave diode laser with an excitation wavelength of 405 nm and an output power of 0.7 mW. A variable neutral density filter was used to attenuate the output power of the laser source. The emitted light was collected and focused, via free space optics, onto the entrance slit of a single monochromator equipped with a thermoelectrically cooled CCD detector operating in the 200 –1100 nm wavelength range. A 455 nm long pass filter, placed before the entrance slit, prevented any reflected laser beam from entering the spectrometer. Figure S8 shows the PL set up and Figure S9 shows an irradiated CsPbBr_3_ QD-based PDS.

### PDS fabrication

PDSs were fabricated by depositing the QDs on different substrates by spin coating and dip coating. The spin coating was done in three steps with speeds 200 rpm, 1000 rpm, and 500 rpm to obtain uniform coatings on the substrates. The coatings were dried by heating the substrate to 90 °C followed by a vacuum baking step. The dip-coating speeds were limited to 5 mm per minute. These substrates were also dried using the technique used for spin-coated substrates. TPB was deposited on the PDS using both vapor deposition and solution-based techniques.

### PDS characterization

For the PDS characterization, parts from the Ocean Optics spectroscopy system were used. The Ocean Optics spectroscopy system is comprised of a 35 W Xenon lamp with an output of 185–2000 nm. The Ocean Optics Monoscan2000 is a fiber-optic scanning monochromator that can select excitation wavelengths of 220–700 nm delivered by an optical fiber to the sample. The excitation wavelength light was varied from 240 to 380 nm. SensL C-series SiPMs were used as photodetectors for all the PDS. The SiPM is soldered to a miniaturized preamplifier circuit with unity gain. Figure S11 shows the SiPM and miniaturized SiPM electronics. The BNC output cable is used for testing the SiPM signal using a Keithley 2400 source measure unit (SMU).

## Supplementary information

Supplementary Video 1.

Supplementary Information.
