# Supplementary material for: Highly efficient photon detection systems for noble liquid detectors based on perovskite quantum dots
Source: Sci Rep. 2020 Oct 9;10:16932. doi: 10.1038/s41598-020-73437-x (PMC7547111; doi:10.1038/s41598-020-73437-x)
Supplement: Supplementary file 2 — Supplementary Information. [file 41598_2020_73437_MOESM2_ESM.pdf]

Highly Efficient Photon Detection Systems For Noble Liquid Detectors Based On Perovskite Quantum Dots

A. Datta,[1, a)] B. Barman,[2] S. Magill,[3] S. Motakef [1]

[1]CapeSym, Inc., 6 Huron Drive, Natick, MA 01760

[2]University of Michigan, 303 E Kearsley St, Flint, MI 48502

[3]Argonne National Laboratory, 9700 S Cass Ave, Lemont, IL 60439

Supplementary Material

Table S1. $CsPbX_3$-based detector configurations that were tested in addition to $CsPbBr_3$ QDs.

**QD Chemical Composition**	**Synthesis Procedure, Temperature**	**Typical PLQY (%)**	**Emission Wavelength (nm)**
**$CsPbCl_{1.5}Br_{1.5}$**	OAm synthesis, 160ºC	30.95	442
	OAm synthesis, 170ºC	28.40	443
	OAm synthesis, 190ºC	25.20	443
**$CsPbCl_{1.5}Br_{1.5}$ + $CsPbBr_3$**	OAm synthesis, 170ºC & OAm synthesis, 155ºC	49.03	497
	OAm synthesis, 170ºC & OAm synthesis, 190ºC	47.21	499
**$CsPbCl_3$**	OAm synthesis, 170ºC	38.63	403
	OAm synthesis, 170ºC, YCl_3-treated	2.79	409
	OAm synthesis, 170ºC, $CdCl_2$-treated	10.04	405

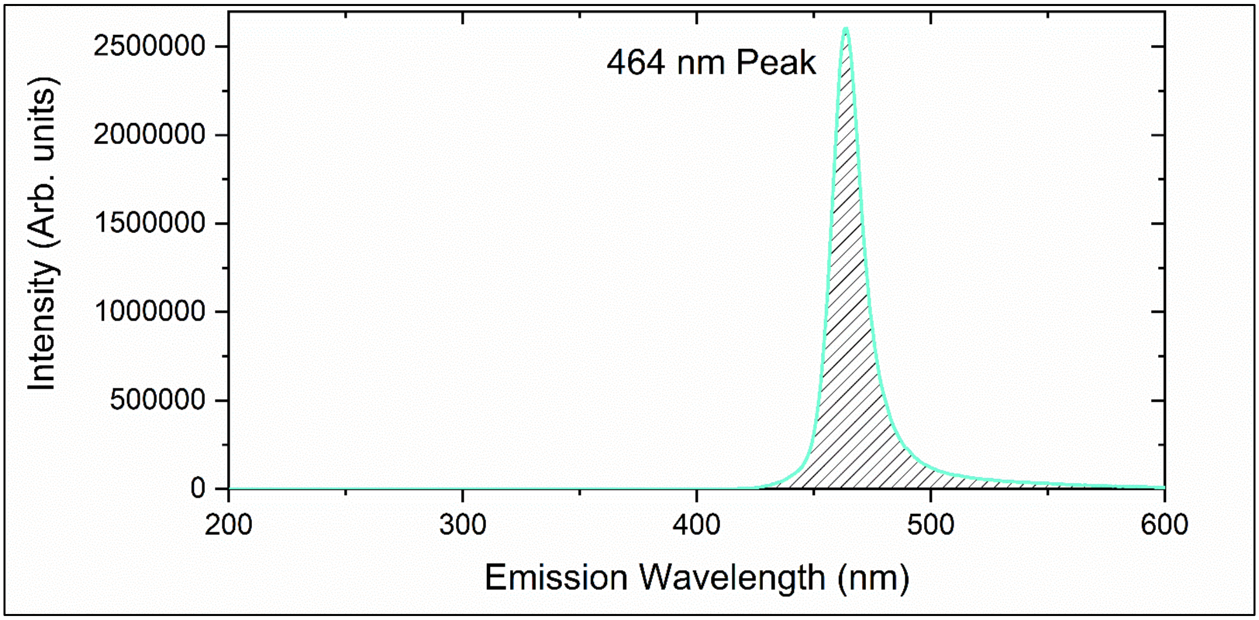

Figure S1 Emission Spectrum of $CsPbBr_{1.5}Cl_{1.5}$ QDs synthesized at 190ºC.

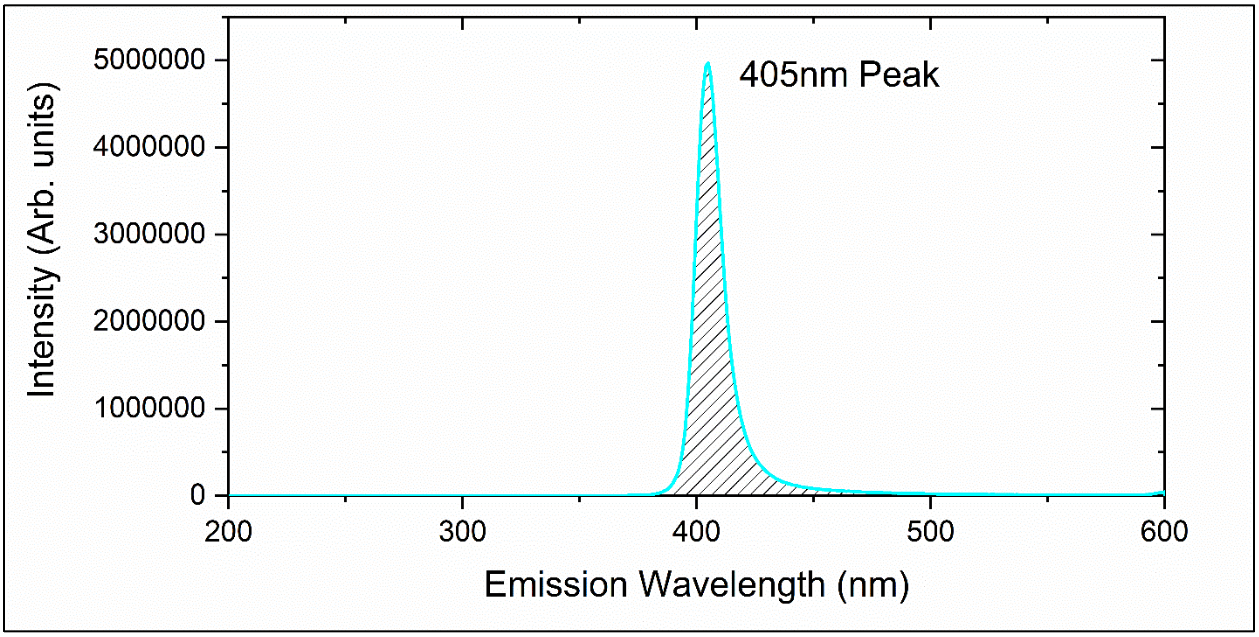

Figure S2 Emission Spectrum of $CsPbCl_3$ QDs synthesized at 170ºC.

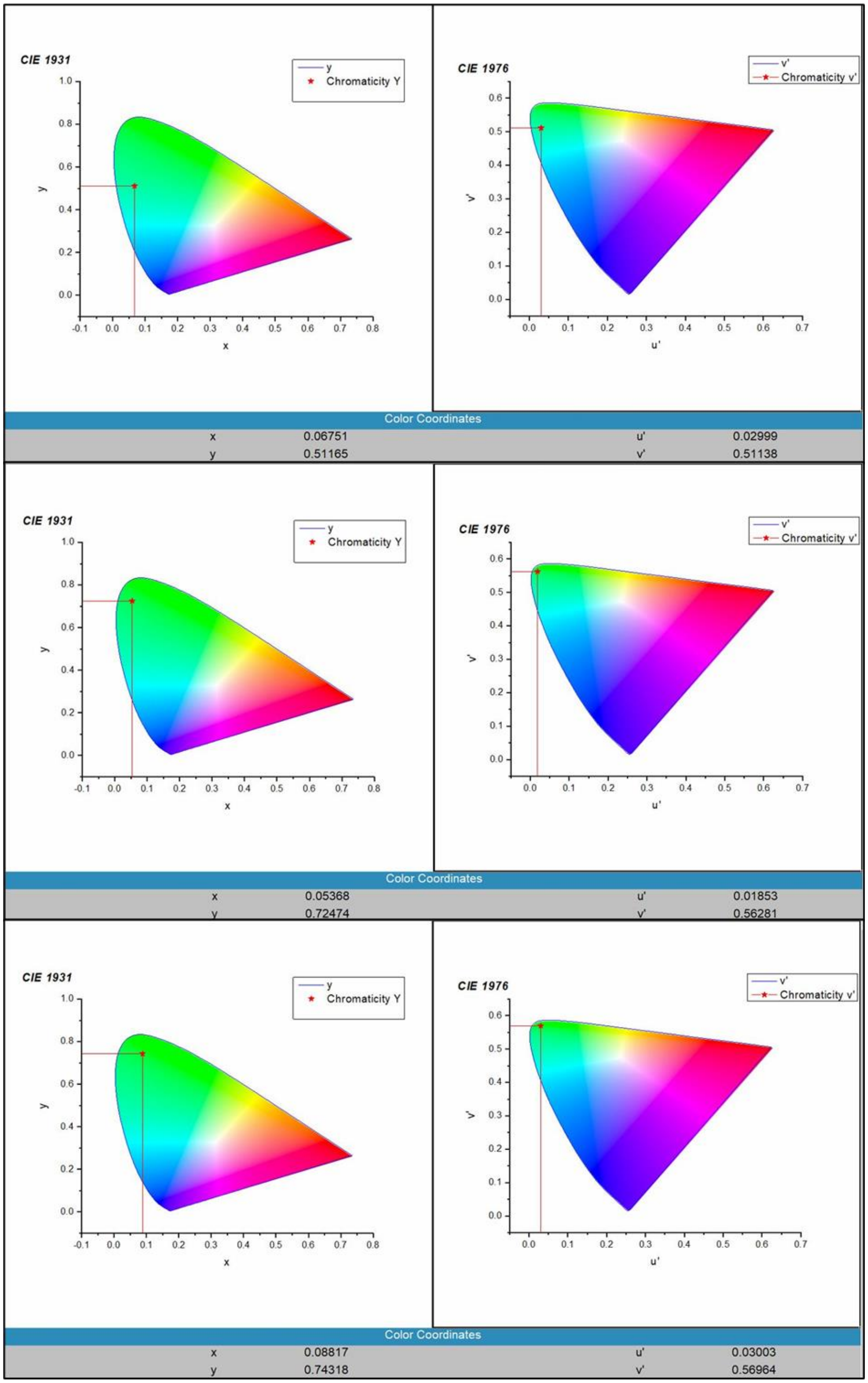

Figure S3 Color Gamut of the $CsPbBr_3$ synthesized using the OAm technique at 155ºC, 180ºC and 190ºC (top to bottom).

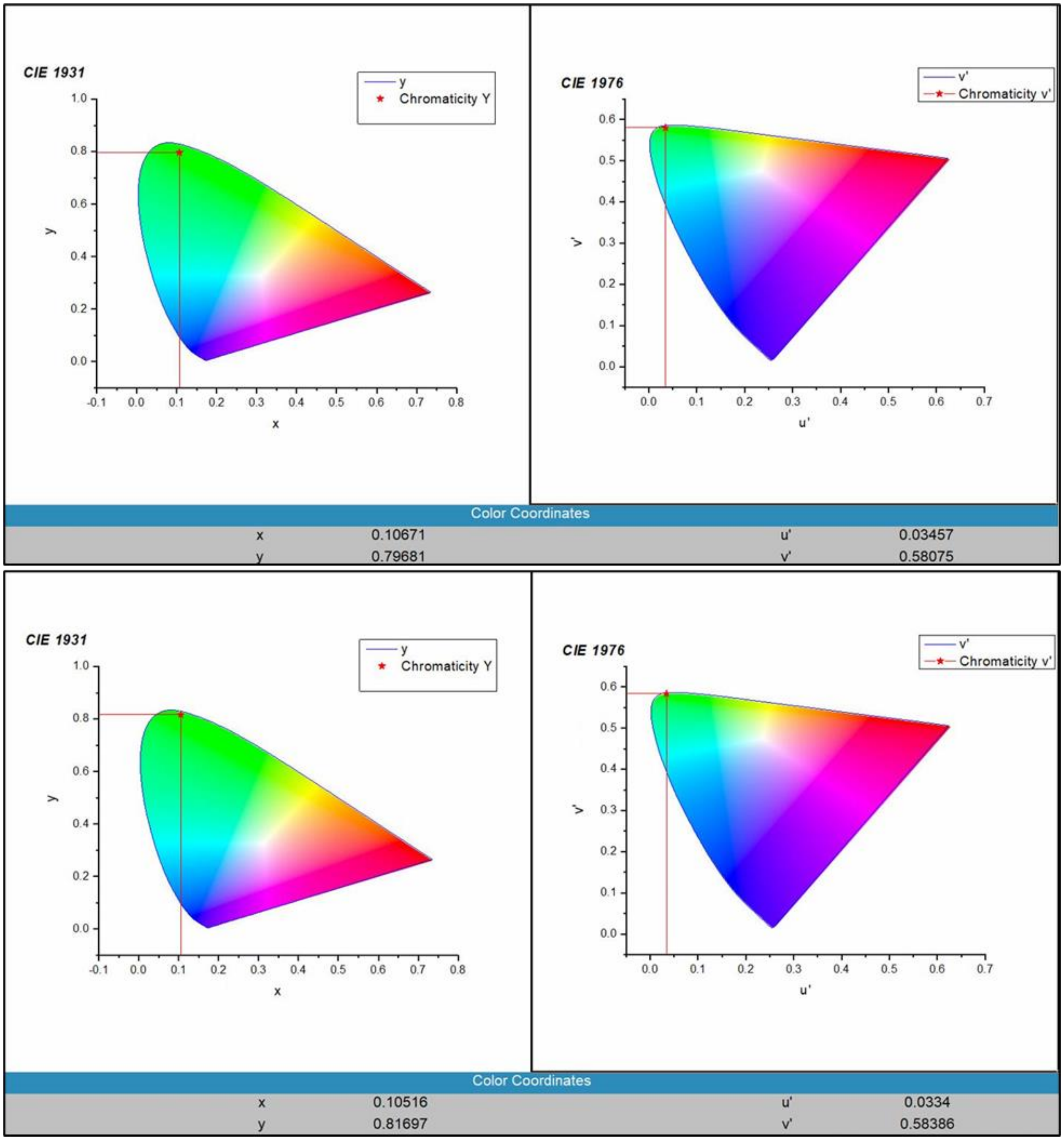

Figure S4 Color Gamut of the $CsPbBr_3$ synthesized using the DBSA technique under stoichiometric and Pb-excess precursor conditions (top to bottom).

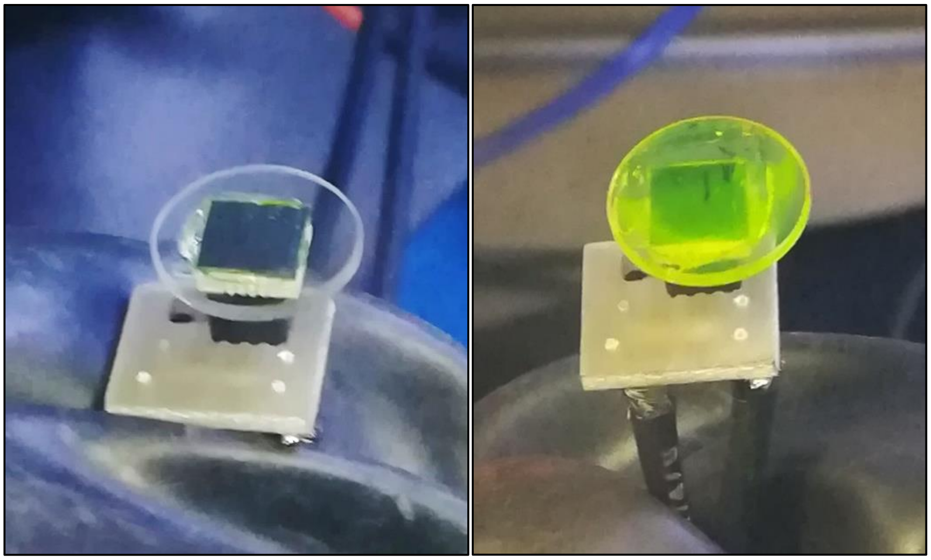

Figure S5 Silica-based $CsPbBr_3$ QD PDS attached to a SiPM, control at left and QD-coated on the right.

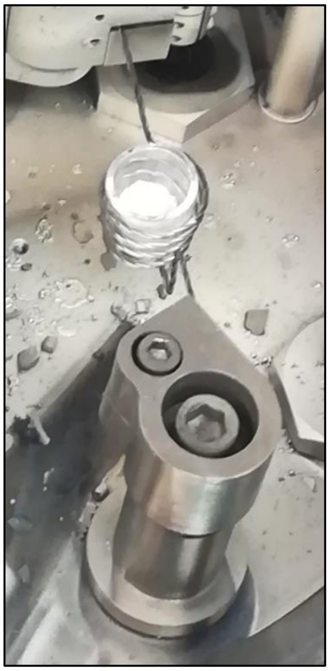

Figure S6 TPB source material in a quartz cup for thermal evaporation inside an Argon glovebox.

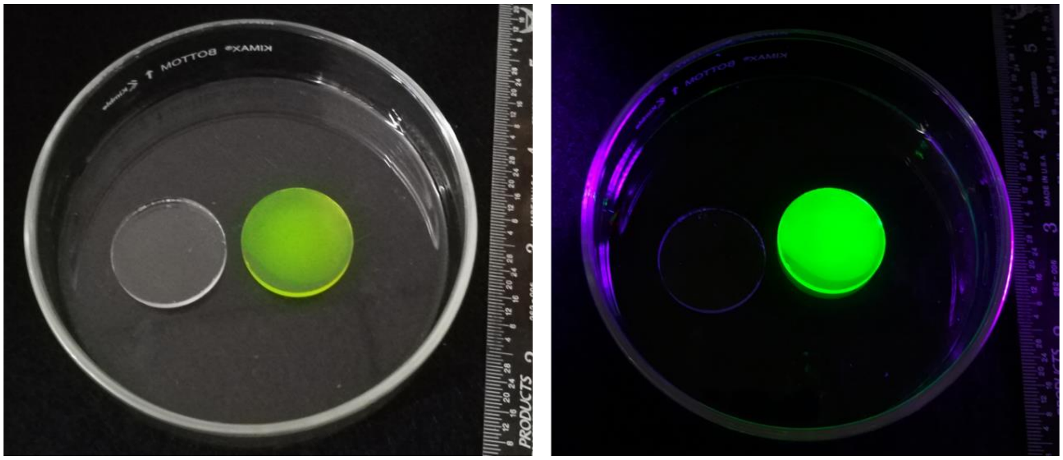

Figure S7 Uncoated and $CsPbBr_3$-QD coated 25.5mm diameter acrylic disc under (left) ambient light and (right) UV light.

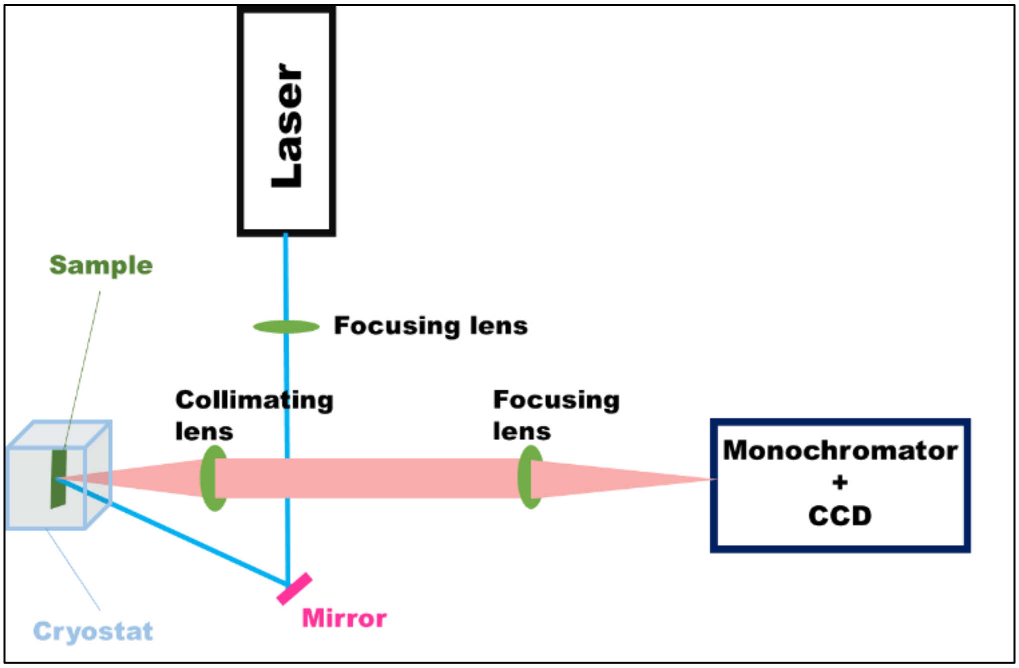

Figure S8 Experimental set up for PL study of $CsPbBr_3$ QDs.

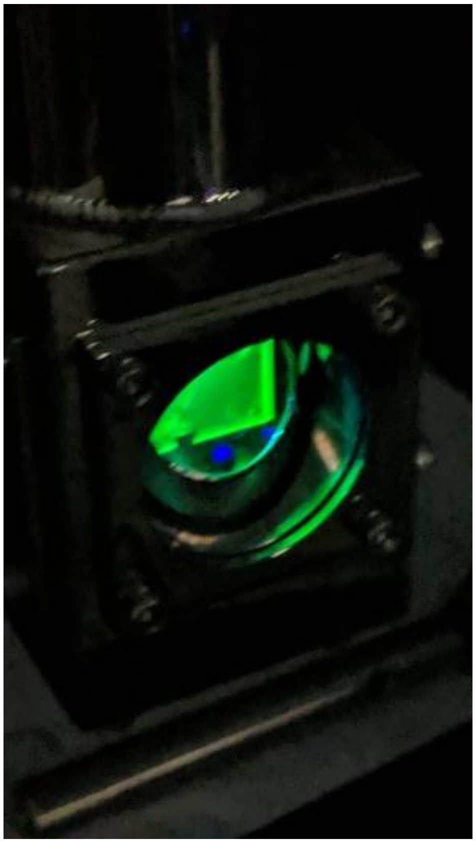

Figure S9 Irradiated $CsPbBr_3$ QD-based PDS inside the PL set up.

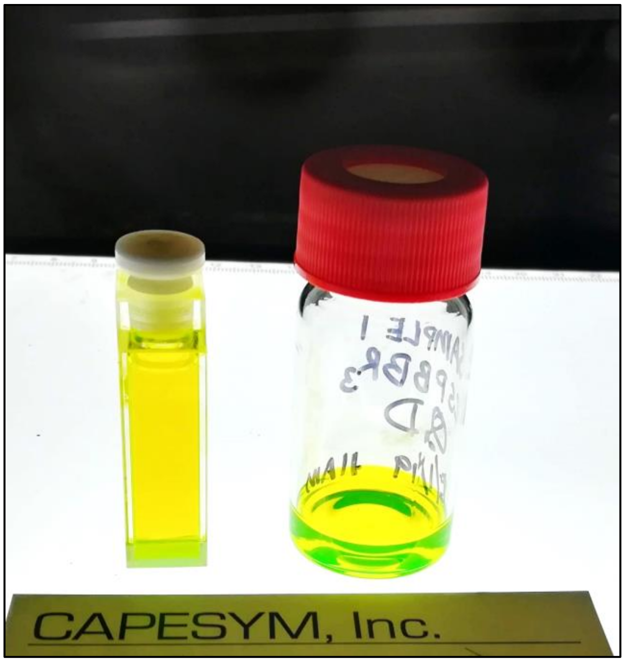

Figure S10 $CsPbBr_3$ colloidal solution in hexane.

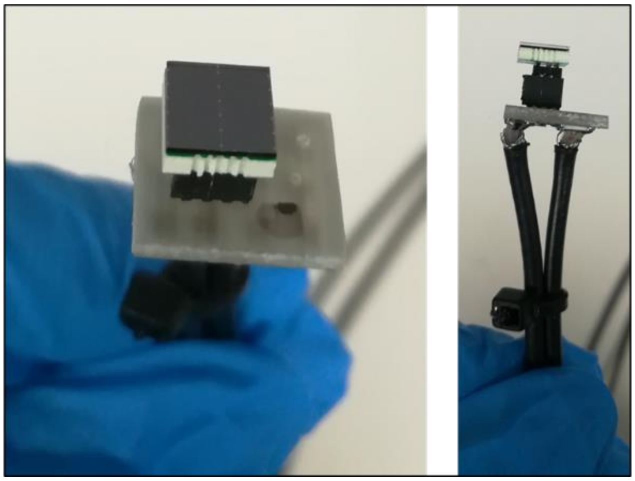

Figure S11 6mm x 6mm area SenSL SiPM with miniaturized low-noise unity gain pre-amplifier circuit connected to a BNC output cable.
